# Succession of lignocellulolytic bacterial consortia bred anaerobically from lake sediment

**DOI:** 10.1111/1751-7915.12338

**Published:** 2016-02-15

**Authors:** Elisa Korenblum, Diego Javier Jiménez, Jan Dirk van Elsas

**Affiliations:** ^1^ Department of Microbial EcologyGroningen Institute for Evolutionary Life SciencesUniversity of GroningenGroningenThe Netherlands; ^2^ Department of Plant and Environmental SciencesWeizmann Institute of ScienceRehovot76100Israel

## Abstract

Anaerobic bacteria degrade lignocellulose in various anoxic and organically rich environments, often in a syntrophic process. Anaerobic enrichments of bacterial communities on a recalcitrant lignocellulose source were studied combining polymerase chain reaction–denaturing gradient gel electrophoresis, amplicon sequencing of the 16S rRNA gene and culturing. Three consortia were constructed using the microbiota of lake sediment as the starting inoculum and untreated switchgrass (*Panicum virgatum*) (acid or heat) or treated (with either acid or heat) as the sole source of carbonaceous compounds. Additionally, nitrate was used in order to limit sulfate reduction and methanogenesis. Bacterial growth took place, as evidenced from 3 to 4 log unit increases in the 16S rRNA gene copy numbers as well as direct cell counts through three transfers on cleaned and reused substrate placed in fresh mineral medium. After 2 days, *Aeromonas bestiarum‐*like organisms dominated the enrichments, irrespective of the substrate type. One month later, each substrate revealed major enrichments of organisms affiliated with different species of *Clostridium*. Moreover, only the heat‐treated substrate selected *Dysgonomonas capnocytophagoides‐*affiliated bacteria (Bacteroidetes). Towards the end of the experiment, members of the Proteobacteria (*Aeromonas*,*Rhizobium* and/or *Serratia*) became dominant in all three types of substrates. A total of 160 strains was isolated from the enrichments. Most of the strains tested (78%) were able to grow anaerobically on carboxymethyl cellulose and xylan. The final consortia yield attractive biological tools for the depolymerization of recalcitrant lignocellulosic materials and are proposed for the production of precursors of biofuels.

## Introduction

Lignocellulose is naturally depolymerized by enzymes of microbial communities that develop in soil as well as in sediments of lakes and rivers (van der Lelie *et al*., 2012). Sediments in organically rich environments are usually waterlogged and anoxic, already within a centimetre or less of the sediment water interface. Therefore, much of the organic detritus is probably degraded by anaerobic processes in such systems (Benner *et al*., 1984). Whereas fungi are well‐known lignocellulose degraders in toxic conditions, due to their oxidative enzymes (Wang *et al*., 2013), in anoxic environments bacteria may be the main plant biomass degraders.

Lignocellulose feedstocks, such as agricultural and forest residues, can be used to produce a wide range of value‐added bioproducts (e.g. biogas, enzymes, antioxidants) and biofuels (Bhatia *et al*., 2012; Peacock *et al*., 2013). Current approaches that use lignocellulose waste for biofuel production are still economically nonviable and hence improvement in biodegradation rates is dearly needed (Banerjee *et al*., 2010). The structure of lignocellulose, which is mainly composed of cellulose, hemicellulose and lignin (Bhatia *et al*., 2012), represents a constraint for its biodegradability. Lignin is very stable and it also ties/shields off the polysaccharide chains, which explains the recalcitrance of lignocellulose to bioconversion. Previous studies have shown that (chemical and/or physical) pretreatment increases lignocellulose breakdown, by loosening the bonds between the lignin and the polysaccharide moieties, to such an extent that the glycoside bonds are easier accessed by enzymes (Ahring and Westermann, 2007; Kumar and Murthy, 2011).

The use of bacterial consortia able to degrade (hemicellulose), with a focus on anaerobic ones, appears to represent a viable strategy to enhance biodegradation rates. However, and rather surprisingly, studies on the structure and composition of lignocellulolytic communities are rarely conduct under anoxic conditions. A study of switchgrass‐degrading anaerobic bacteria, enriched from tropical forest soils, revealed dominant organisms to consist of members of the Firmicutes, Bacteroidetes and Alphaproteobacteria (DeAngelis *et al*., 2012). Another study, which enriched bacteria from sugarcane bagasse compost under aerobic (static) conditions, revealed the co‐occurrence of two dominant anaerobic genera, *Clostridium* and *Thermoanaerobacterium*, together with aerobic bacilli next to as‐yet‐uncultured bacteria (Wongwilaiwalin *et al*., 2010). Previous studies have also enriched microorganisms on different plant biomass along successive transfers (Brossi *et al*., 2015; Porsch *et al*., 2015). For instance, some of these enrichments were designed to favour anaerobic fermentation (methanogenesis) with the concomitant production of biomethane from pretreated wheat straw (Sträuber *et al*., 2015).

However, the improvement might be limited to initial stages of degradation using a single bacterial culture, and the (hemi)cellulose degradation rate of pretreated lignocellulose decreases along consecutive transfers (He *et al*., 2013). To overcome biomass recalcitrance to degradation, applications of mixed bacterial cultures, in which succession takes place, is likely critical to the biodegradation of complex polymers (Fierer *et al*., 2010). In order to enhance the prevalence of degraders and boost bacterial succession, in this study, recalcitrance was addressed by reusing (i.e. recycling) lignocellulose in consecutive transfers of anaerobic bacterial consortia. We established three consortia consisting of anaerobically growing bacteria bred from lake sediment on treated (HSG and ASG) or untreated switchgrass (USG) as the sole sources of carbon and energy. The three enrichment cultures were designed to allow anaerobic respiration by nitrate reduction given the added potassium nitrate. The presence of the strong electron acceptor nitrate apparently will not strongly inhibit fermentative metabolism, but it does preclude methanogenesis and sulfate reduction (Chen *et al*., 2008; Oren, 2009). The dynamics of the phylogenetic composition and abundance of the bacterial communities developing on the recalcitrant biomass is described. In addition, we confirmed the (hemi)cellulolytic activities of isolated members of the communities, which were able to grow anaerobically on carboxymethyl cellulose (CMC) and xylan.

## Results

### Establishment of lignocellulose substrate‐adapted bacterial consortia

**Figure 1:**
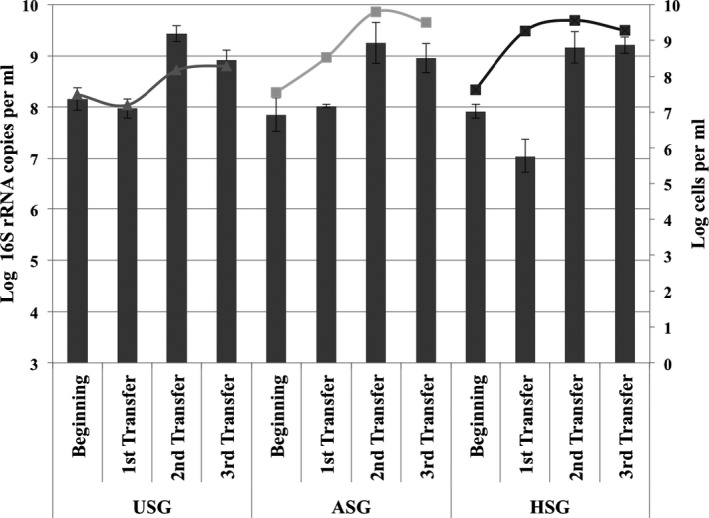
Bacterial cell counts (lines) and 16S rRNA gene copies (bars) of enriched cultures..

Bacterial growth took place in the three successive transfers in the media containing USG, ASG and HSG as the sole carbon source, as well as in the second and third transfers where lignocellulosic substrates were reused (Fig. 1). Two to three log unit increases of cell densities were evidenced by total microscopic cell counts as well as qPCR of the 16S rRNA gene after 2 days (denoted ‘Beginning’) and 1 month of the first transfer. Density increases were also detected when the partially biodegraded switchgrass from the first transfer was reused for the second and third transfers. During 1 month of anaerobic incubation, no growth was observed in blank (control) flasks (without substrate) after the successive transfers (data not shown). Denaturing gradient gel electrophoresis (DGGE) profiles of the PCR‐amplified 16S rRNA gene revealed that diverse bacterial communities had been enriched by the three treatments from the lake sediment inoculum. Moreover, the consortia changed over time in all differentially treated systems (Fig. S1). The PCR‐DGGE profiles formed well‐defined clusters with high similarity among replicates, as confirmed by a permutation test (*P* < 0.05).

### Succession of bacterial taxa growing on lignocellulose substrate under anoxic conditions

Amplicon pyrosequencing (based on the bacterial 16S rRNA gene) of consortium DNA of the three replicate USG, ASG and HSG samples at four time points produced a total of 71 305 reads (mean: 1981 per sample, range 639–4518) after quality trimming, while the sediment sample yielded 18 944 cleaned reads (Table 1). Rarefaction analysis suggested that the coverage of the respective consortia was generally sufficient (Fig. S2). Analysis of all sequences across all bacterial enrichment cultures over time revealed the presence of a total of 724 operational taxonomic units (OTUs) (97% nucleotide identity cut‐off), while the original sediment sample showed the presence of 1125 OTUs (Table 1, Table S1). Only 53 OTUs were shared among all enriched consortia (Fig. S3).

**Table 1:** 16S rRNA amplicon libraries after quality control

	Sediment	USG				ASG				HSG			
		B	1st	2nd	3rd	B	1st	2nd	3rd	B	1st	2nd	3rd
No. of sequences (min–max)	18 944	1241–1692	639–1417	1040–1929	1368–2801	4208–4518	1749–2304	2428–2631	1902–3893	914–1256	817–2373	2073–2394	1067–3124
**OTUS**	**1125**	**195**	**126**	**158**	**150**	**200**	**184**	**141**	**114**	**194**	**164**	**166**	**96**
PD	8.43	1.53	1.75	1.66	1.64	1.17	1.63	1.34	0.94	1.99	2.23	2.4	1.51
Chao‐1	188.68	40.94	57.55	39.58	45.61	63.33	51.46	51.11	32.00	59.83	44.50	36.30	38.56

**Figure 2:**
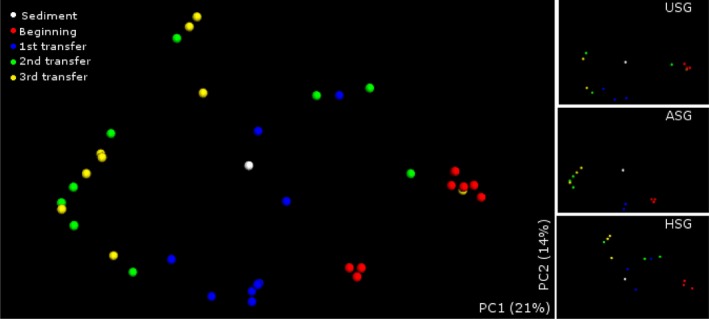
PCoA ordination plot shows relatedness of all samples over time using unweighted UniFrac distance of classified 16S rRNA gene sequence (0.97 similarity). PCoA of sample distances shows principal coordinate 1 (PC1) and principal coordinate 2 (PC2) with a total of 35% of variation explained. The right small panels depict the PCoA ordination of sediment sample with samples USG, ASG or HGS separately.

Faith's phylogenetic diversity (PD) and Chao richness (Chao‐1) estimators changed in all three enrichment cultures over time (Table 1; Fig. S4). The HSG systems revealed the highest PD values at the first and second transfers. Thus, particular bacterial populations were consistently selected throughout the transfers and the consortia encompassed phylogenetically more related species over time. Principal coordinate analysis (PCoA) of unweighted pairwise UniFrac distances between samples depicted a similar pattern of distribution along PC1 and PC2 (35% of variation explained), clustering the bacterial consortia over time (Fig. 2). However, the pairwise analysis of similarity (ANOSIM) testing of the unweighted pairwise UniFrac distances confirm that substrate pretreatment was a key factor driving consortium dissimilarity over time (*R* = 0.6676; *P* < 0.0001; Table S2).

**Figure 3:**
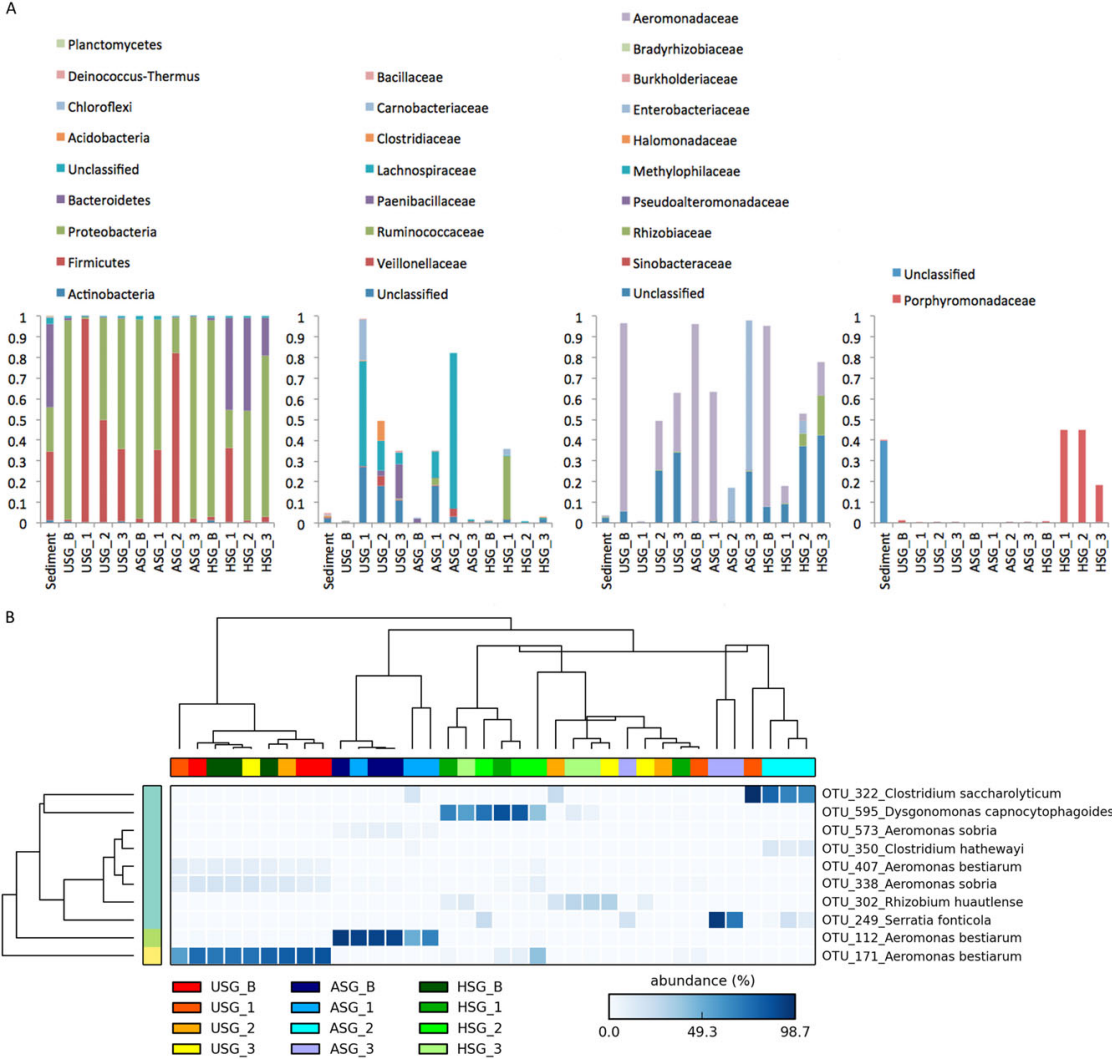
A. Relative abundance at phylum (first graph) and family levels based on 16S rRNA gene amplicon sequencing in each consortia over time. B. Heat map of the relative abundance of the 10 most abundant OTUs composing more than 1% differently enriched in samples (ANOVA, FDR,* P* < 0.05).

Taxonomic analysis of the communities demonstrated the dominance of organisms from five phyla in all samples, i.e. Proteobacteria, Firmicutes, Bacteroidetes, Actinobacteria and Acidobacteria. Proteobacteria and Firmicutes accounted for most of the sequences, representing 96, 99 and 67% of all sequences in the USG, ASG and HSG systems, respectively (Fig. 3A). In addition, Bacteroidetes were highly enriched (31%) in HSG. At lower taxonomic rankings, OTUs belonging to Actinobacteria and Acidobacteria had less than 2% relative abundance across all consortia. The bacterial families found to dominate the switchgrass consortia were minorities in the sediment community (Table S3). The dominating Porphyromonadaceae (Bacteroidetes) and Rhizobiaceae (Alphaproteobacteria) occurred at 0.3 and 0.16%, respectively, whereas Lachnospiraceae, Carnobacteriaceae (Firmicutes), Aeromonadaceae and Enterobacteriaceae (Gammaproteobacteria) represented less than 0.1% of the total sediment bacterial community (Fig. 3A; Table S3). In the ‘beginning’, all consortia were dominated by Gammaproteobacteria, with sequences related to Aeromonadaceae amounting to ca. 90% (Fig. 3A; Table S3). Two OTUs (OTU_112 and OTU_171; Fig. 3B), identified as *Aeromonas bestiarum*, were significantly enriched, OTU_112 representing 38–87% of reads in ASG consortia in the beginning and in the end of the first transfer, and OTU_171 >50% of total reads in the beginning of USG and HSG consortia [analysis of variance (ANOVA), *P* < 0.05, false discovery rate (FDR)]. As the consortia developed in the second transfer, a shift to different families of Firmicutes (Lachnospiraceae) was noted in ASG and USG, while in HSG Firmicutes (Ruminococcaceae) and Bacteroidetes (Porphyromonadaceae) dominated. The OTU_595 identified as *Dysgonomonas capnocytophagoides* was highly enriched in HSG only, while the OTU_322, identified as *Clostridium saccharolyticum*, was strongly associated with samples USG and ASG (ANOVA, *P* < 0.05, FDR). Each of these OTUs represented >50% of the reads per sample. At the end of the third transfer, a dominance of Gammaproteobacteria was found across all three consortia. The OTU_302, identified as *Rhizobium huautlense*, was significantly enriched in the USG and HSG consortia, and the OTU_249, affiliated with *Serratia fonticola*, was predominant in ASG consortia (ANOVA, *P* < 0.05, FDR).

Some sequences of the libraries were only identified as belonging to the domain Bacteria in USG (2538 reads, 15.6%), ASG (680 reads, 1.9%) and HSG (2897 reads, 15.6%); these represented ca. one‐third of all OTUs of the three consortia (254 OTUs).

### Isolation and characterization of (hemi)cellulolytic bacterial strains

Colonies with different morphologies detected after 48 h of growth at 25°C under anaerobic conditions from the plated samples of the USG‐, ASG‐ and USG‐adapted consortia were selected. Thus 42, 69 and 49 bacterial strains were obtained, respectively (total of 160 isolates, Table S4), and their 16S rRNA gene sequence determined (Table S5). Analyses using BLAST‐N revealed, across all isolates, similarities of more than 97% to sequences from known bacteria. Fourteen bacterial species were identified in total, with five being highly abundant in the 16S rRNA amplicon libraries when OTUs were identified with BLAST‐N using the NCBI database (Tables S6 and S7), i.e. *A. bestiarum*,* Clostridium algidixylanolyticum*,* C. saccharolyticum*,* Clostridium xylanolyticum* and *D. capnocytophagoides*. Moreover, the remaining strains were also found in the amplicon libraries, in lower amounts (*Ensifer adhaerens*,* Rhizobium daejeonense*,* Serratia proteamaculans*,* Sporotalea propionica*,* Actinotalea fermentans*) or they remained undetected (*Clostridium beijerinckii*,* Cellulomonas cellasea*,* Isoptericola hypogeus* and *Pseudoclavibacter soli*). Fifty bacterial strains were randomly taken to test their abilities to grow on cellulose and (hemi)celluloses using CMC and xylan from beechwood as the carbon sources in minimal medium agar under anoxic conditions. A total of 39 isolates (78%) were found to grow on both substrates, producing a degradation halo around the colony, as evidenced by iodine staining (Table 2; Fig. S6). Twenty‐three isolates belonging to Firmicutes, five *S. propionica* and 18 *Clostridium* isolates, could grow on CMC and xylan. Among the 14 Proteobacteria that degraded (hemi)cellulose, five were *A. bestiarum*, seven *E. adhaerens* and two *S. proteamaculans* isolates. One out of four actinobacteria, identified as *C. cellasea*, was able to grow on these substrates. Finally, one out of three Bacteroidetes, i.e. *D*. *capnocytophagoides*, grew on cellulose and (hemi)cellulose (Table 2).

**Table 2:** (Hemi)cellulose degrading bacteria: growth on glucose, fructose, CMC and xylan of selected isolates under anoxic conditions

	Isolate strains	Identification (blast)	Growth		(Growth/activity)	
			Glucose 0.2%	Fructose 0.2%	CMC 0.2%	Xilan 0.2%
USG_B	Ci7	*Clostridium algidixylanolyticum*	**+**	**+**	**(+/+)**	**(+/+)**
	Ci11	*Clostridium saccharolyticum*	**+**	**+**	**(+/+)**	**(+/+)**
	Ci23	*Clostridium saccharolyticum*	**+**	**+**	**(+/+)**	**(+/+)**
	Ci18	*Clostridium xylanolyticum*	**+**	**+**	**(+/+)**	**(+/+)**
	Ci9	*Clostridium xylanolyticum*	**+**	**+**	**(+/+)**	**(+/+)**
USG_3	cBf1	*Clostridium saccharolyticum*	**+**	**+**	**(+/+)**	**(+/+)**
	cBf13	*Clostridium saccharolyticum*	**+**	**+**	**(+/+)**	**(+/+)**
	CCf1	*Clostridium saccharolyticum*	**+**	**+**	**(+/+)**	**(+/+)**
ASG_B	ai10	*Aeromonas bestiarum*	**+**	**+**	**(+/+)**	**(+/+)**
	ai17	*Aeromonas bestiarum*	**+**	**+**	**(+/+)**	**(+/+)**
	ai18	*Aeromonas bestiarum*	**+**	**+**	**(+/+)**	**(+/+)**
	ai22	*Aeromonas bestiarum*	**+**	**+**	**(+/+)**	**(+/+)**
	ai7	*Aeromonas bestiarum*	**+**	**+**	**(+/+)**	**(+/+)**
	ai9	*Aeromonas bestiarum*	**W**	**−**	**(−/−)**	**(−/−)**
	ai8	*Clostridium algidixylanolyticum*	**+**	**+**	**(+/+)**	**(+/+)**
	ai3	*Clostridium beijerinckii*	**+**	**+**	**(+/+)**	**(+/+)**
	ai4	*Clostridium beijerinckii*	**+**	**+**	**(+/+)**	**(+/+)**
	ai13	*Clostridium xylanolyticum*	**+**	**+**	**(+/+)**	**(+/+)**
	ai14	*Clostridium xylanolyticum*	**W**	**−**	**(−/−)**	**(−/−)**
	ai19	*Clostridium xylanolyticum*	**+**	**+**	**(+/+)**	**(+/+)**
	ai2	*Serratia proteamaculans*	**+**	**+**	**(+/+)**	**(+/+)**
	ai21	*Serratia proteamaculans*	**+**	**+**	**(+/+)**	**(+/+)**
	ai1	*Sporotalea propionica*	**+**	**+**	**(+/+)**	**(+/+)**
	ai5	*Sporotalea propionica*	**W**	**−**	**(−/−)**	**(−/−)**
	ai6	*Sporotalea propionica*	**+**	**+**	**(+/+)**	**(+/+)**
ASG_3	aBf15	*Cellulomonas cellasea*	**+**	**+**	**(+/+)**	**(+/+)**
	aBf6	*Cellulomonas cellasea*	**W**	**−**	**(−/−)**	**(−/−)**
	aAf1	*Clostridium saccharolyticum*	**+**	**+**	**(+/+)**	**(+/+)**
	aAf3	*Clostridium xylanolyticum*	**+**	**+**	**(+/+)**	**(+/+)**
	aCf11	*Clostridium xylanolyticum*	**+**	**+**	**(+/+)**	**(+/+)**
	aCf13	*Clostridium xylanolyticum*	**W**	**−**	**(−/−)**	**(−/−)**
	aBf10	*Ensifer adhaerens*	**+**	**+**	**(+/+)**	**(+/+)**
	aBf12	*Ensifer adhaerens*	**+**	**+**	**(+/+)**	**(+/+)**
	aBf30	*Ensifer adhaerens*	**+**	**+**	**(+/+)**	**(+/+)**
HSG_B	Hi10	*Clostridium algidixylanolyticum*	**+**	**+**	**(+/+)**	**(+/+)**
	Hi15	*Clostridium saccharolyticum*	**+**	**+**	**(+/+)**	**(+/+)**
	Hi24	*Sporotalea propionica*	**+**	**+**	**(+/+)**	**(+/+)**
	Hi4	*Sporotalea propionica*	**+**	**+**	**(+/+)**	**(+/+)**
	Hi6	*Sporotalea propionica*	**W**	**−**	**(−/−)**	**(−/−)**
HSG_3	HBf5	*Actinotalea fermentans*	**W**	**−**	**(−/−)**	**(−/−)**
	HBf8	*Actinotalea fermentans*	**W**	**−**	**(−/−)**	**(−/−)**
	HA18	*Dysgonomonas capnocytophagoides*	**W**	**−**	**(−/−)**	**(−/−)**
	HB15	*Dysgonomonas capnocytophagoides*	**+**	**+**	**(+/+)**	**(+/+)**
	HCf7	*Dysgonomonas capnocytophagoides*	**W**	**−**	**(−/−)**	**(−/−)**
	HA10	*Ensifer adhaerens*	**+**	**+**	**(+/+)**	**(+/+)**
	HA24	*Ensifer adhaerens*	**+**	**+**	**(+/+)**	**(+/+)**
	HB1	*Ensifer adhaerens*	**+**	**+**	**(+/+)**	**(+/+)**
	HC10	*Ensifer adhaerens*	**+**	**+**	**(+/+)**	**(+/+)**
	HCf16	*Isoptericola hypogeus*	**W**	**−**	**(−/−)**	**(−/−)**
	HA9	*Sporotalea propionica*	**+**	**+**	**(+/+)**	**(+/+)**

(+) indicates positive growth or halo of degradation of CMC or xylan; (−) indicates no growth; (**W**) indicates weak growth.

## Discussion

The production of sugar monomers from lignocellulose substrate requires pretreatment of feedstock due to its recalcitrance to microbial degradation (Guo *et al*., 2011). Therefore, in this study, we assessed the dynamics of bacterial taxa in three enrichment cultures bred from a sediment sample on untreated, heat‐ or acid‐treated switchgrass (denoted as USG, HSG and ASG, respectively) under anoxic conditions. These substrates were recycled in consecutive transfers and the bacterial successions over three transfers were assessed on the basis of PCR‐DGGE as well as bacterial 16S rRNA pyrosequencing. Given that a large fraction of natural microbial communities is not detected using culture‐dependent techniques (Staley and Konopka, 1985), we opted for these direct molecular approaches. Combination of these with cultivation might also shed light on initially ‘rare’ organisms (Pedrós‐Alió, 2012). Interestingly, the sequencing data revealed that a suite of bacteria that were in low RA (0.6%) in the sediment did grow in the experimental systems (Fig. 3, Table S2). In the USG, ASG and HSG systems, such organisms overwhelmed the populations that were initially abundant in the sediment. Our conditions were set so as to favour the use of nitrate as the terminal electron acceptor, to the detriment of that of iron, manganese, sulfate or fermentation and the production of methane (Londry and Suflita, 1999). Clearly, in natural sediments, methane is often produced from lignocellulose as the major final product upon depletion of electron acceptors such as nitrate, iron, manganese and sulfate (Ahring and Westermann, 2007). The reasoning to exclude these groups was based on the potential to use the bacterial consortia that were bred for bioethanol production, in which case the presence of sulfide and methane would be undesirable. However, some bacteria performing fermentative metabolism may have been favoured by the three switchgrass enrichments.

In the first transfer, disregarding the substrate used in the enrichment, the most dominant OTUs were related to *A. bestiarum*, which might have grown on lignocellulose (either treated or untreated) by anaerobic respiration with nitrate. Consistent with this assumption is the fact that genes encoding nitrate (and nitrite) reductases are often present in the core genome of members of the genus *Aeromonas* in MetaRef (clade 1231; Huang *et al*., 2014) and the reference genome of the *A. bestiarum* species in RefSeq collection of NCBI (protein table 16435_217332). Moreover, the *A. bestiarum* strains obtained were able to grow on xylan and CMC; these activities presumably allowed their growth at the beginning on fresh untreated and pretreated substrates.

In this study, substrate recycling was applied to strongly select for bacterial consortia that are best able to perform degradation, establishing the type of degradation dynamics found in nature (Peacock *et al*., 2013). After the first switchgrass breakdown step, the dominant OTUs were related to *C. saccharolyticum* (in USG and ASG) and *D. capnocytophagoides* (in HSG), indicating a change of metabolic status in the enrichments. Here, one might surmise that fermentative processes became more dominant as compared with anaerobic respiration with nitrate. The relative dominance of these species may also indicate their proneness to ‘work’ under the conditions applied (i.e. already partially degraded substrate), under which they flourish, with the levels of nitrate present (Murray, 1986). The community structures of these consortia were influenced by the carbon source, being the HSG cultures more diverse than the USG and ASG ones. The bacterial populations, at the highest taxonomic level, showed shifts, with members of the Proteobacteria (mainly *A. bestiarum*) alternating with those of the Firmicutes on USG and ASG; or Bacteroidetes in the HSG consortia. Bacteroidetes were found with high frequency in the HSG consortia only, indicating the competitiveness of this group in HSG systems. During thermal processes, both hemicellulose and lignin are solubilized, which may release easily degradable compounds such as acetate, formate, next to furfural (Trifonova *et al*., 2008; Hendriks and Zeeman, 2009). Previously, we showed that Bacteroidetes may also play a role in furfural uptake (Jiménez *et al*., 2014). Substances released during the heat treatment might be at the basis of the difference in the bacterial consortia in HSG from the USG or ASG. In contrast, acid pretreatment mainly solubilizes hemicellulose, which could explain that the USG‐ and ASG‐adapted cultures shared similar phylogenetic traits. Recently, we (Jiménez *et al*., 2014) assessed aerobic soil‐derived microbial consortia bred on heat‐treated and untreated wheat straw as the lignocellulosic source material. PCR‐DGGE applied to these systems showed that substrate pretreatment did affect the bacterial and fungal consortia that were selected.

Members of the consortia related to *C. saccharolyticum* or *D. capnocytophagoides* degraded the recalcitrant biomass, and then, at the third transfer, OTUs related to *Serratia* and *Rhizobium* dominated the enrichment cultures. These bacteria were, thus, mainly active on the switchgrass, previously ASG and HSG, which were already partially degraded. Members of *Serratia* and *Rhizobium* occur typically in systems such as the rhizosphere (Wang *et al*., 1998; Carneiro *et al*., 2013), where cellulases or hemicellulases may be important for survival; both types are nitrate reducers.

To a lesser extent, Actinobacteria were found in the three enrichments. Thirteen isolates were obtained, and these were affiliated with *A. fermentans*,* C. cellasea*,* I. hypogeus* and *P. soli*. These phyla are frequently found in cellulolytic processes, such as in compost, soil and the termite hindgut (Warnecke *et al*., 2007; DeAngelis *et al*., 2011; Sizova *et al*., 2011). Finally, given the fact that fungi are regarded as the main decomposers of plant residues under aerobic conditions (Bugg *et al*., 2011), we assessed their presence by PCR of the ITS1 region. This only yielded evidence for fungal presence in the ASG adapted consortia only after the first transfer (data not shown). Thus, the degradation of switchgrass under anaerobiosis such as applied by us was indeed mainly orchestrated by bacterial consortia that act by anaerobic respiration and/or fermentation. A shift in dominance from *A. bestiarum* to *C. saccharolyticum* or *D. capnocytophagoides* and then to *R*. *huautlense* or *S*. *fonticola* was found, and this is tentatively related to the progressively increasing recalcitrance of the substrate. In addition, when the switchgrass is pretreated by heat, *D. capnocytophagoides* plays an important role in the resulting consortium. Thus, consortia consisting of degraders with different energy generation strategies (fermentation and nitrate reduction) were constructed, which potentially relates to different enzymatic batteries and efficiencies. Concerning application, synthetic microbial communities that lack sugar monomer scavengers need to be formulated using the isolates obtained and evaluated in a bioreactor as to the metabolic efficiencies in polymer deconstruction.

## Experimental procedures

### Lignocellulose material and sediment sampling

Air‐dried switchgrass were cut into pieces of 2 cm in length. Then, 20 g of this material was used to acid and heat pretreatments. The acid treatment was performed by soaking the biomass in 500 ml of 1 M acetic acid under static condition at room temperature for 24 h, then washed with tap water until it reach a neutral pH and oven‐dried (Guo *et al*., 2011). The heat treatment was conducted by the torrefaction of the biomass at 200°C for 1h (Trifonova *et al*., 2008). USG‐, ASG‐ and HSG‐treated biomasses were ball milled into 2 mm of fibres. These lignocellulosic substrates were added separately as carbon source in mineral medium described below to enrich lignocellulolytic facultative anaerobic cultures. The same sediment sample was used as starting bacterial inoculum of all three consortia and it was collected from a lake in the city of Groningen, The Netherlands (53°14′44.9376″N; 6°32′4.0236″E). The sediment was obtained below 1.5 m of water column depth. The temperature at the sampling point was 8°C and sediment suspension in water was near neutral pH (7.3). Total cell counts were performed from sediment suspension diluted decimally.

### Enrichment cultures and bacterial community analysis

The mineral medium was prepared as follows (g l^−1^): K_2_HPO_4_, 1.0; NaCl, 1.0; MgSO_4_.7H_2_O, 0.1; NH_4_Cl, 2.0; CaCl_2_, 0.1; NaHCO_3_, 0.35; NaNO_3_, 0.22, the final pH was adjusted to 7.2. Flasks with rubber sealing lid and closed with screw caps containing 250 ml of mineral medium and 2.5 g of ball‐milled switchgrass (pretreated or raw) were autoclaved. A total of nine enrichments were prepared: three flasks using raw switchgrass as carbon source, three for ASG and the other three for HSG. Additional filter‐sterile solutions were introduced using a sterile syringe: metal solution, 0.3 ml; vitamin solution, 0.3 ml; resazurin solution 0.1%, 1 ml; sodium thioglycolate 1.5%, 3 ml. Metal solution was composed of (g l^−1^) EDTA, 0.5; Co(NO_3_), 0.1; MnSO_4_.H_2_O, 0.5; AlK(SO_4_)_2_, 1.0; CaCl_2_.2H_2_O, 0.1; ZnSO_4_.7H_2_O, 0.1; FeSO_4_.7H_2_O, 0.1; CuSO_4_.5H_2_O, 0.01; Na_2_MoO_4_.2H_2_O, 0.01; H_3_BO_3_, 0.01; Na_2_SeO_4_, 0.005; NiCl_2_.6H_2_O, 0.003. Vitamin solution contained (mg l^−1^) biotin, 2.0; folic acid, 2.0; pyridoxine‐HCl, 10.0; thiamine‐HCl, 5.0; riboflavin, 5.0; nicotinic acid, 5.0; DL‐calcium pantothenate, 5.0; vitamin B12, 0.1. Sediment contained 10^8^ cells per ml and the same diluted sediment suspension, in sterile phosphate buffer, was used as inoculum and an aliquot was transferred to media using 1 ml syringe, the final cell concentration in each culture was 10^5^ cells per ml. All enrichment cultures were anaerobic (by adding sodium thioglycolate as reducing agent and potassium nitrate, 1.0 g l^−1^, as terminal electron acceptor available; and indicated by resazurin) and were incubated in the dark at 25°C under slow shaking (80 rpm) for 1 month. Following this time period, bacterial suspension of the enrichment cultures was harvested anaerobically using syringe and transferred to empty sterile Vacutainer tubes (BD Biosciences). In order to recycle the lignocellulosic substrate into fresh mineral medium to further culture transfers, the switchgrass fibres were washed thoroughly with distilled water and air‐dried, and therefore the recalcitrant biomass is mainly reintroduced into the vials. The dry biomass was reintroduced to a new flask with fresh mineral medium. After autoclaving, total cell counts were performed from the latter culture and inoculated to its original substrate, at a final concentration of 10^5^ cells per ml. This procedure was repeated twice, and then three culture transfers were carried out. Four samplings were carried out: (i) 2 days after the first transfer denoted as the beginning; (ii) 30 days following the first transfer; (iii) 30 days following the second transfer; and (iv) 30 days after the third transfer. Additionally, three control flasks per substrate without sediment suspension (inocula) were carried out as negative controls. Moreover, blank flasks were carried out with inoculums, but no carbon source added.

### Bacteria quantification

Total bacterial counts of each sample replicates were performed at the end of each transfer using a Neubauer chamber (cell depth: 0.02 mm) on light microscope. In addition, an aliquot (1 ml) from the enrichment cultures and controls was harvested using 1 ml syringe at four sampling time, from which the genomic DNA of the community was extracted and purified using the Fast DNA kit (MP Bio) according to manufacturer's instructions. To estimate bacterial abundance, we used qPCR absolute quantification of 16S rRNA gene, with primers FP (5′‐GGTAGTCYAYGCMSTAAACG‐3′) and RP (5′‐GACARCCATGCASCACCTG‐3′) (Bach *et al*., 2002). Power SYBR Green PCR master mix (Finnzymes, Finland), 0.5 μl BSA (20 mg ml^−1^) 0.8 μM of each primer and 1 μl of template DNA (2–10 ng) of sample or dilution standard per well. Standard curve was prepared by decimally diluting plasmids containing partial 16S rRNA gene amplicons to range from 10^9^ to 10^3^ copy numbers. qPCR cycling detection (264 bp product) was performed as follows: initial denaturation at 95°C for 10 min; amplification for 40 cycles consisting of denaturation at 95°C for 27s, primer annealing at 62°C for 1 min, extension at 72°C for 30s, followed by melting curve analysis to confirm specific PCR products. Reactions were performed in three replicates on an ABI Prism 7300 Cycler (Applied Biosystems).

### DGGE

Bacterial community structure was analysed by 16S rRNA gene PCR‐DGGE approach, as described by Brons and van Elsas (2008). Briefly, primers were F‐968 (5′‐AA CGC GAA GAA CCT TAC‐3′) with clamp (5‐CGC CCG GGG CGC GCC CCG GGC GGG GCG GGG GCA CGG GGG G) and R‐1401 (5′‐CGG TGT GTA CAA GGC CCG GGA ACG‐3′) and denaturing gradient range was 45–65% in 6% gels, samples ran for 16 h at 60°C (100 V). DGGE patterns were compared by clustering the different lanes, using the unweighted pair group method with Dice coefficient using GelCompar II software (Applied Maths, Sint‐Martens‐Latem, Belgium). DGGE patterns were also subjected to permutation test using permtest package of software R (version 2.15.3). Binary data from the DGGE profile were imported into R software (version 2.15.3) with vegan package (version 2.0–4) and BiodiversityR package (version 2.2), a site‐to‐site Bray–Curtis dissimilatory matrix constructed using the vegdist function; site ordinations plotted using the metaMDS and the ordisymbol functions.

### 16S rRNA gene pyrosequencing and data analysis

Bacterial 16S rRNA gene compositions in the three enrichment cultures along four sampling time (n = 3, total 36 enrichment samples) and in the original sediment sample were analysed using a tag‐encoded amplicon pyrosequencing assay, universal primers S‐D‐Bact‐0008‐a‐S‐16 (5′‐AGAGTTTGATCMTGGC‐3′) and S‐D‐Bact‐0907‐a‐A‐20 (5′‐CCGTCAATTCMTTTGAGTTT‐3′) (Klindworth *et al*., 2013) were used that targeted the variable regions V1–V3 of the 16S rRNA gene. A total of 37 samples were multiplexed and ran in a 1/8 plate 454 run. PCR amplification for library construction and pyrosequencing, with Roche 454 FLX instrument and Titanium reagents (Roche/454 Life Sciences), was performed by LGC Genomics, Germany. All 16S rRNA pyrosequencing reads were analysed using QIIME (version 1.7.0, available in Bio‐Linux 7) (Field *et al*., 2006; Caporaso *et al*., 2010), with the default arguments in the split_libraries.py function, after primer trimming. After trimming, sequences were grouped into OTUs; at 97% of nucleotide identity, using a standard QIIME pipeline, we used the UCLUST method in the pick_otus.py function and the RDP method to classify OTUs using the assign_taxonomy.py function. QIIME was also used for alpha diversity analyses (10 sampling repetitions without replacement at each sampling depth) including rarefaction to 500 reads per sample, computation of Chao1 and phylogenetic diversity (PD_whole_tree); and beta diversity analyses, unweighted Unifrac distance matrix was constructed from the phylogenetic tree and visualized using PCoA. ANOSIM was carried out with the R (http://www.R-project.org) examining pairwise UniFrac distances (9999 permutations). SFF files containing the original unfiltered pyrosequences can be downloaded from the NCBI Short Read Archive, accession number: SRP030618. Besides that, the QIIME IDs from each OTU were used to check for common OTUs among treatments in each time period through Venny (Oliveros, 2007). OTUs that represented more than 1% of reads in total per sample were used for hierarchical clustering coupled with heat map analysis based on relative abundance of OTUs that differed significantly, identified via ANOVA with the Tukey–Kramer post‐hoc test (confidence interval of 0.95) and the Benjamini–Hochberg multiple test FDR correction using the STAMP (Statistical Analysis of Metagenomic Profiles) software (Parks and Beiko, 2010).

#### Identification of (hemi)cellulolytic anaerobic bacteria isolated from enrichment cultures

After the first and third transfers, bacteria were isolated from each enrichment culture on R2A agar supplemented with NaNO_3_ 0.2 g l^−1^. All plates were incubated in AnaeroJars using the system Anaerocult (Merck) to generate gas to anaerobiosis incubation. The jars were incubated at 25°C for 1 week before isolating individual colonies. Colonies were selected randomly and streaked on R2A with NaNO_3_ and again incubated under anoxic conditions, until purity was reached. Enzymatic activity tests for (hemi)cellulases were carried out in minimum agar (g l^−1^): NaNO_3_, 2.0; K_2_HPO_4_, 1.0; MgSO_4_, 0.5; KCl, 0.5; peptone, 0.2; agar, 17.0) using 2 g l^−1^ as a final concentration of CMC, xylan, glucose or fructose, (Jiménez *et al*., 2014). A drop (20 μl) of bacterial culture grown anaerobically overnight was inoculated on agar plate containing CMC, xylan, glucose or fructose. Plates were incubated at 28°C for 72 h under anaerobic conditions (using Anaerocult, Merck) and flooded with iodine (33% of I_2_ and 67% of KI) (Kasana *et al*., 2008) to reveal the hydrolysis halo on the agar plate. Plates containing fructose or glucose, used to evaluate anaerobic bacterial growth, were flooded with iodine. These plates were used as negative controls to evaluate CMC and xylan degradation assay (Jiménez *et al*., 2014). Bacterial 16S rRNA genes were amplified by colony PCR approach. Briefly, a small amount of pure colony was transferred to 50 μl of 50 mM NaOH solution using a toothpick and submitted to heat at 95°C for 15 min. Then, 3 μl of each cell lysate was added to a PCR premix tube, and PCR was performed using the following primers: 27F (5′‐AGAGTTTGATCMTGGCTCAG‐3′) and 1492R (5′‐GGTTACCTTGTTACGACTT‐3′). Amplification was performed under the following conditions: initial denaturation at 94°C for 5 min; 35 cycles of 94°C for 30s, 52°C for 30s, 72°C for 1 min; and a final extension step at 72°C for 10 min. Positive PCR products were purified using the Promega Wizard PCR Clean‐Up System (Promega). The purified PCR products were sequenced with the 27F primer, by LGC Genomics company, Germany, which generated high‐quality sequences of ca. 800 bp. Sequences were deposited in GenBank with accession numbers KJ194837–KJ194996. The sequences were identified using a local BLAST search against the pre‐formatted 16S microbial database (ftp://ftp.ncbi.nih.gov/blast/db/16SMicrobial.tar.gz) using blast‐2.2.22 + (http://blast.ncbi.nlm.nih.gov/Blast.cgi) with default settings. OTUs were also identified using a local BLAST search against the pre‐formatted 16S microbial database using blast‐2.2.22 + with default settings, in order to associate the taxonomic identification of the isolates.

## Conflict of interest

The authors declare no conflict of interest.
